# Supplementary material for: COZOID: contact zone identifier for visual analysis of protein-protein interactions
Source: BMC Bioinformatics. 2018 Apr 6;19:125. doi: 10.1186/s12859-018-2113-6 (PMC5889581; doi:10.1186/s12859-018-2113-6)
Supplement: Supplementary file 2 — Software build. Executable binary file of the software tool. (ZIP 118,784 kb) [file 12859_2018_2113_MOESM2_ESM.zip › cozoid/java/docs/beansbinding-1.2.1-doc.zip!/org/jdesktop/observablecollections/ObservableListListener.html]

ObservableListListener (Beans Binding)


---


|  |  |  |
| --- | --- | --- |
| **Overview** \| **Package** \| **Class** \| **Use** \| **Tree** \| **Deprecated** \| **Index** \| **Help** | |  |
| **PREV CLASS**   **NEXT CLASS** | **FRAMES**    **NO FRAMES**     **All Classes** |  |
| SUMMARY: NESTED \| FIELD \| CONSTR \| METHOD | DETAIL: FIELD \| CONSTR \| METHOD |  |


---


## org.jdesktop.observablecollections Interface ObservableListListener

**All Superinterfaces:**: java.util.EventListener

**All Known Implementing Classes:**: ListBindingManager

---

``` public interface ObservableListListener extends java.util.EventListener ```

Notification types from an `ObservableList`.

---

| **Method Summary** | |
| --- | --- |
| `void` | `listElementPropertyChanged(ObservableList list, int index)`             Notification than a property of an element in this list has changed. |
| `void` | `listElementReplaced(ObservableList list, int index, java.lang.Object oldElement)`             Notification that an element has been replaced by another in the list. |
| `void` | `listElementsAdded(ObservableList list, int index, int length)`             Notification that elements have been added to the list. |
| `void` | `listElementsRemoved(ObservableList list, int index, java.util.List oldElements)`             Notification that elements have been removed from the list. |

**Method Detail**

### listElementsAdded

```
void listElementsAdded(ObservableList list,
                       int index,
                       int length)
```

:   Notification that elements have been added to the list.

    :   **Parameters:**: `list` - the `ObservableList` that has changed: `index` - the index the elements were added to: `length` - the number of elements that were added

---


### listElementsRemoved

```
void listElementsRemoved(ObservableList list,
                         int index,
                         java.util.List oldElements)
```

:   Notification that elements have been removed from the list.

    :   **Parameters:**: `list` - the `ObservableList` that has changed: `index` - the starting index the elements were removed from: `oldElements` - a list containing the elements that were removed.

---


### listElementReplaced

```
void listElementReplaced(ObservableList list,
                         int index,
                         java.lang.Object oldElement)
```

:   Notification that an element has been replaced by another in the list.

    :   **Parameters:**: `list` - the `ObservableList` that has changed: `index` - the index of the element that was replaced: `oldElement` - the element at the index before the change

---


### listElementPropertyChanged

```
void listElementPropertyChanged(ObservableList list,
                                int index)
```

:   Notification than a property of an element in this list has changed.
    Not all `ObservableLists` support this notification. Only
    observable lists that return `true` from
    `supportsElementPropertyChanged` send this notification.

    :   **Parameters:**: `list` - the `ObservableList` that has changed: `index` - the index of the element that changed


---


|  |  |  |
| --- | --- | --- |
| **Overview** \| **Package** \| **Class** \| **Use** \| **Tree** \| **Deprecated** \| **Index** \| **Help** | |  |
| **PREV CLASS**   **NEXT CLASS** | **FRAMES**    **NO FRAMES**     **All Classes** |  |
| SUMMARY: NESTED \| FIELD \| CONSTR \| METHOD | DETAIL: FIELD \| CONSTR \| METHOD |  |


---
